# Preventing Thyroid Eye Disease with Statins: Evidence from a Meta-Analysis

**DOI:** 10.7759/cureus.84388

**Published:** 2025-05-19

**Authors:** Maxim J Barnett, Tara A John, Carlo Casipit, Sarah Eidbo, Justin Lam, Catherine Anastasopoulou

**Affiliations:** 1 Internal Medicine, Jefferson-Einstein Hospital, Philadelphia, USA; 2 Endocrinology, Diabetes and Metabolism, Jefferson-Einstein Hospital, Philadelphia, USA

**Keywords:** active thyroid eye disease, grave's ophthalmopathy, graves' orbitopathy, statin use, thyroid eye disease

## Abstract

Statins, a class of medications that inhibit 3-hydroxy-3-methylglutaryl coenzyme A reductase, are the first-line treatment for cholesterol reduction. Beyond their anti-lipidemic properties, statins have gained recent attention for their anti-inflammatory effects. Recently, it has been shown that when prescribed early in the course of Graves’ disease, there appears to be a reduction in the progression toward thyroid eye disease. This study aims to provide an overall estimate of the effect of statin therapy in relation to the development of thyroid eye disease by conducting a meta-analysis. Both CINAHL and MEDLINE databases were used to screen for studies without a time-restricted period. Studies had to include a control (no statin therapy) and treatment group (received statin therapy) with patients at baseline harboring a diagnosis of Graves’ disease. Studies had to provide a hazard (or odds) ratio (HR or OR) and 95% confidence intervals, which were subsequently combined using the generic inverse variance method. Six studies were ultimately included in the meta-analysis (from an initial n = 28 studies identified). A pooled meta-analysis demonstrated a significantly reduced hazard ratio of developing thyroid eye disease in patients treated with statin therapy (HR 0.57; 95% CI 0.44-0.74, p < 0.05). Significant heterogeneity was present in our study (I^2^ = 87.8%). This is the first meta-analysis to provide an overall estimate of the beneficial effects of statins toward preventing the development of thyroid eye disease in patients with Graves’ disease. The exact mechanism remains unknown but is believed to be an extra-lipidemic manifestation, relating to the anti-inflammatory properties of the statin medications. Further studies are required to confirm our findings and identify optimal candidates for early initiation of statin therapy in Graves’ disease.

## Introduction and background

Statins, first discovered in 1976 by Japanese microbiologist Akira Endo, are potent inhibitors of 3-hydroxy-3-methylglutaryl coenzyme A reductase, preventing the endogenous synthesis of cholesterol [1]. The first statin, mevastatin, was discovered from the fungus *Penicillium citrinum* [1]. Statins are grouped by intensity into low, medium, and high subgroups. Apart from their anti-lipid properties, numerous extra-cholesterol manifestations of statins have been documented within the literature, with benefits including (but not limited to) reduction in certain types of cancer (such as prostate), prevention of neurodegenerative diseases (such as Alzheimer’s), limitation in developing contrast-induced nephropathy, and anti-inflammation. Elevated cholesterol levels are believed to be pro-inflammatory; however, other direct effects of statins are thought to play a part in reducing inflammation. Such examples include reduced inflammatory markers (such as C-reactive protein), cytokines inhibition (tumor necrosis factor alpha and interferon gamma), and prevention of T-helper cell migration [2]. The anti-inflammatory effects are believed to be statin-specific (irrespective of cholesterol levels) and have not been reproduced with other anti-lipid classes of agents [2].

A particular area of uncertainty regarding the anti-inflammatory properties of statins involves thyroid eye disease (Graves’ ophthalmopathy). We previously reported a retrospective analysis and literature review describing such preventative effects of statins in thyroid eye disease [3]. This study further explores this correlation by analyzing the current literature and performing a meta-analysis to provide an estimate of the overall effect.

## Review

Search strategy

Both CINAHL and MEDLINE were used for an advanced search to identify relevant papers without a time restriction by four investigators (CC, TJ, SE, and JL). Search string included the following terms: “statin”, “thyroid eye disease”, “graves ophthalmopathy”, “graves orbitopathy”, and “exophthalmos”. All studies were assessed for inclusion eligibility and discussed with senior investigators (MB and CA). For our study, we used PRISMA (Preferred Reporting Items for Systematic reviews and Meta-Analyses) methodology [4].

Eligibility criteria

All observational study types were considered for eligibility (case-control, cohort, cross-sectional, and randomized controlled trials), including both abstracts (if applicable) and published, original research. Inclusion criteria required studies to have both a control (not receiving statin therapy) and an experimental group (receiving statin medication) with both groups sharing a common underlying diagnosis of Graves’ disease. Included studies were required to report outcomes of interest regarding thyroid eye disease (odds ratio (OR), relative risk or hazard ratio (HR), and 95% confidence intervals (CI)). There was no geographical or language restriction, but only published work was accepted. Exclusion criteria included papers that failed to provide an OR, HR or relative risk (or failed to include corresponding 95% confidence intervals), did not have a comparator group (not receiving statin therapy), did not define or confirm the diagnosis of Graves’ disease in either group, did not specifically assess outcomes related to thyroid eye disease, involved animal (or in vitro) subjects (rather than humans), or had insufficient methodological detail (which would not allow for assessment of study quality or risk of bias). Additionally, articles such as reviews/editorials/case reports/conference proceedings/commentaries with no original data were excluded.

Statistical analysis

Means, effect sizes, and standard deviations were extracted from each patient group that had been reported. A random-effects model was used due to the heterogeneous population used in the varying studies, alongside differing protocols. DerSimonian and Laird’s generic inverse variance method was incorporated for point estimates and standard errors from each included study. Data was analyzed with a forest plot delivered via an online platform [5]. A funnel plot was used to assess for publication bias, and Egger’s regression test was additionally incorporated to investigate for asymmetry. A trim-and-fill analysis would be incorporated to address publication bias (if identified). Heterogeneity was assessed by Cochrane’s Q test and I^2^ statistics, the latter categorized as follows (0-25%: Insignificant; 26-50%: Low; 51-75%: Moderate; > 75%: High).

Data extraction

Data extracted included a standardized method of author(s), year of publication, country of study origin, population, follow-up duration, intervention, total number of participants, and study design (Table 1) [3, 6-10]. When both unadjusted and adjusted values were reported (HR or OR), the adjusted value was used for data collection and analysis. 

**Table 1:** Characteristics of Included Studies Adapted from Barnett and Colleagues [3]; studies included [3, 6-10]

Authors	Year	Country	Population	Follow-Up	Intervention	Cases and Controls	Study Design	Outcome	Findings
Barnett et al. [3]	2025	Global	Newly diagnosed Graves’ Disease, Age > 18	5 years	All statins (not specified)	Cases: 43, 025 Controls: 43, 025	Retrospective cohort	Thyroid eye disease development	HR 0.56 (95% CI 0.52-0.60) for the development of thyroid eye disease.
Chou et al. [6]	2025	Taiwan	Graves’ Disease, Aged > 40	4.4 years	All statins	Cases: 7, 073 Controls: 95, 785	Retrospective cohort	Thyroid eye disease development	HR 0.64 (95% CI 0.51-0.79) for developing Graves’ Ophthalmopathy
Nilsson et al. [7]	2021	Sweden	Newly diagnosed Graves’ Disease, Age > 18	4.5 years	Simvastatin or atorvastatin	Cases: 5, 574 Controls: 34, 409	Retrospective cohort	Thyroid eye disease development	HR 0.87 (0.76-1.00) for the development of thyroid eye disease.
Hsu et al. [8]	2024	Taiwan	Newly diagnosed Graves’ Disease, Age > 20	62.6 months	Statin (Type not specified)	Cases: 372 Controls: 3, 206	Retrospective cohort	Thyroid eye disease development	OR 0.2 (95% CI 0.08-0.5) for development of thyroid eye disease among statin users.
Lee et al. [9]	2023	Korea	Newly Diagnosed Graves’ Disease	102 months for women	All statins (Not specified)	Cases (Female): 293 Control (Female): 5, 047	Retrospective cohort	Thyroid eye disease development	In women, higher statin dose is associated with reduced risk of thyroid eye disease (HR 0.37, 95% CI 0.22-0.62).
Stein et al. [10]	2015	USA	Newly diagnosed Graves’ Disease, Age > 18	5.6 years	Statin (Type not specified)	Cases: 740 Controls: 7, 664	Retrospective cohort	Thyroid eye disease development	HR 0.60 (95% CI 0.37-0.93) with statin usage for > 60 days over the past year.

Cohort studies were assessed by the Newcastle-Ottawa scale to assess recruitment quality, comparability between groups, and accurate outcome assessments; a score of seven or greater suggested high quality (Table 2) [11]. 

**Table 2:** Newcastle-Ottawa Scale for Included Studies Obtained from references [3, 6-10].

Authors	Year Published	Newcastle-Ottawa Scale	Overall Score
Barnett et al. [3]	2025	Selection: 4, Comparability: 3, Outcome: 3	Overall Score: 8 (High Quality)
Chou et al. [6]	2025	Selection: 4, Comparability: 2, Outcome: 3	Overall Score: 9 (High Quality)
Nilsson et al. [7]	2021	Selection: 4, Comparability: 1, Outcome: 3	Overall Score: 8 (High Quality)
Hsu et al. [8]	2024	Selection: 4, Comparability: 1, Outcome: 3	Overall Score: 8 (High Quality)
Lee et al. [9]	2023	Selection: 4, Comparability: 1, Outcome: 3	Overall Score: 9 (High Quality)
Stein et al. [10]	2015	Selection: 4, Comparability: 1, Outcome: 3	Overall Score: 8 (High Quality)

Results

**Figure 1:**
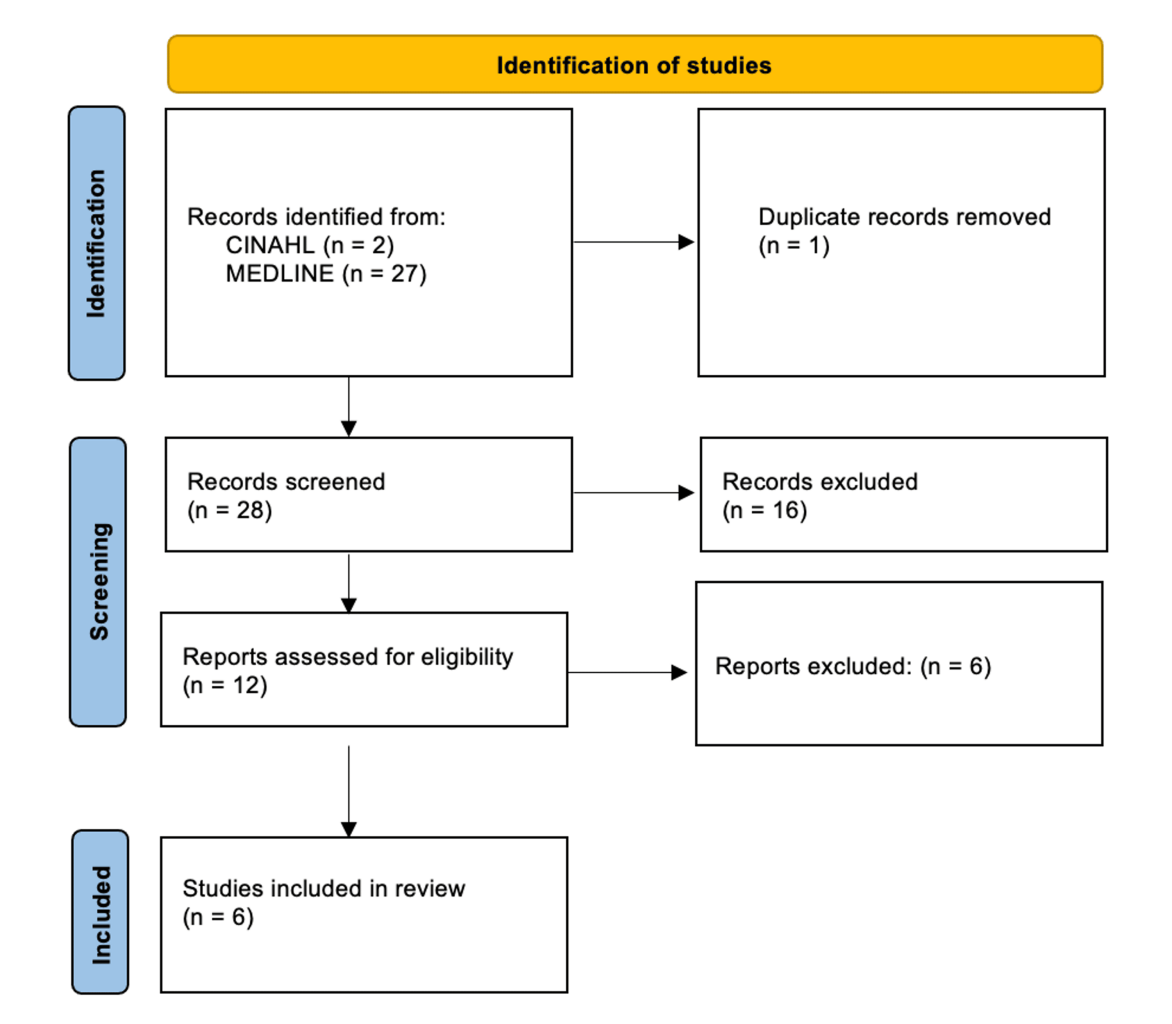
PRISMA Search Strategy

An initial n = 2 studies were identified from CINAHL, and another n = 27 from MEDLINE (n = 1 duplicate was removed). Of the remaining n = 28, n = 22 failed to meet eligibility criteria from the screening title and abstract and were excluded. A final n = 6 studies were included in the meta-analysis [3, 6-10] (Figure 1).

**Figure 2:**
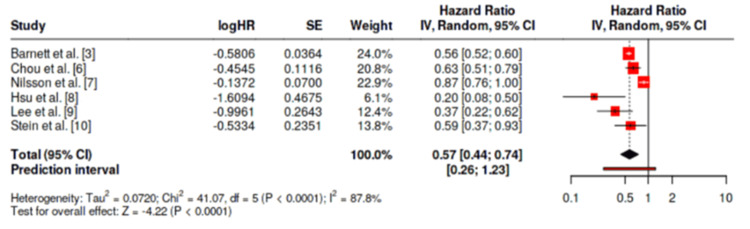
Forest Plot of Included Studies Providing Overall Estimate of Effect Obtained from collation of studies [3, 6-10], Forest Plot created from Fekete and Győrffy [5].

**Figure 3:**
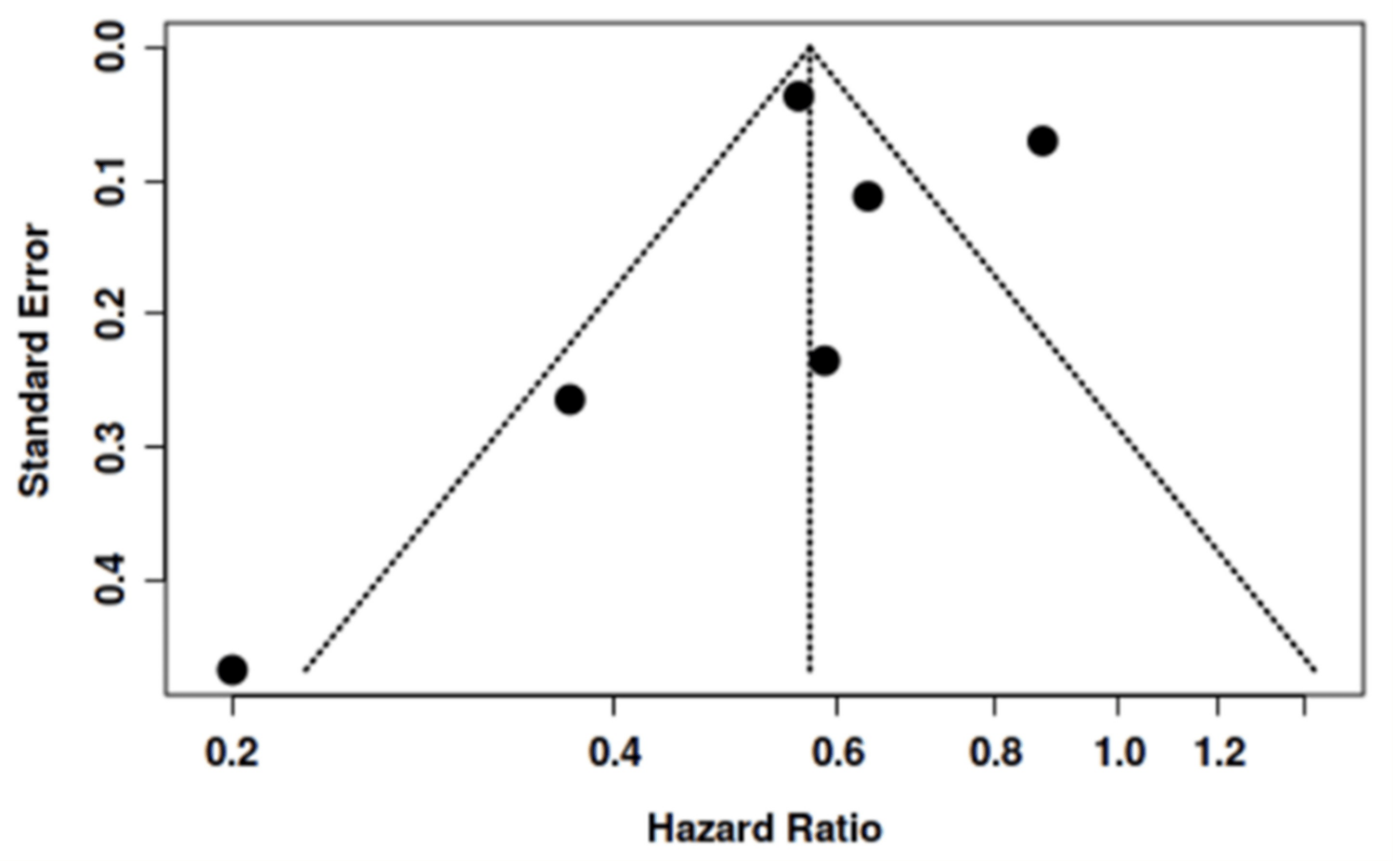
Funnel Plot to Assess Risk of Publication Bias Analyzed studies [3, 6-10], Funnel Plot created from Fekete and Győrffy [5].

In total, n = 6 studies were included in the meta-analysis, with the outcome in question the effect of statin medication on the prevention of thyroid eye disease. The pooled analysis demonstrated an HR of 0.57 (95% CI 0.44-0.74, p < 0.05) for the prevention of thyroid eye disease with statin administration in those with Graves’ disease (Figure 2). High heterogeneity (I^2^ = 87.8%) was present in this study, suggesting variability among the studies is from heterogeneity over random chance. A funnel plot was included, which did not demonstrate a potential for publication bias (Figure 3). Moreover, asymmetry was not supported by Egger’s test (p = 0.817).

As the study by Lee and Colleagues [9] only included women, it was hypothesized that the heterogeneity would decrease to insignificant levels with the removal of this study, however, the I2 increased by 1.5% with the removal of the study. A jackknife sensitivity analysis was subsequently performed with the removal of each study one by one to assess the impact on heterogeneity and investigate if a single study had an exceptionally high influence on between-study variation (Table 3). Nilsson et al. [7] appeared to have the greatest impact on heterogeneity, decreasing to moderate heterogeneity with the removal of their study (I^2^ 53.6%).

**Table 3:** Jackknife Sensitivity Analysis for Heterogeneity Included studies [3, 6-10].

Study Removed	I^2^ Without Study	Change in I^2^
Barnett et al. (2025) [3]	82.7%	-5.1%
Chou et al. (2025) [6]	90.2%	+2.4%
Nilsson et al. (2021) [7]	53.6%	-34.2%
Hsu et al. (2020) [8]	88.7%	+0.9%
Lee et al. (2020) [9]	89.3%	+0.6%
Stein et al. (2015) [10]	90.3%	+2.5%

Discussion

This is the first meta-analysis to provide an overall estimate for the likelihood of progressing toward thyroid eye disease in patients treated with statins for newly diagnosed Graves’ disease. Similar to other available data, these findings continue to reflect the anti-inflammatory properties of statins, extending beyond lipidemic benefits. Notably, one study was excluded from our meta-analysis as it did not provide data on the prevention of thyroid eye disease, but rather analyzed those with pre-existing thyroid eye disease, noting a reduction in decompression or surgery for strabismus [12]. Similarly, only one randomized controlled trial exists, but this too was excluded as the patients were provided with both statins and intravenous glucocorticoids (which differed from the other protocols of the included studies); nonetheless, the randomized controlled trial demonstrated a significant reduction in composite clinical outcomes [13]. We have previously performed a systematic review analyzing the literature about statin therapy and thyroid eye disease [3].

As discussed previously, there appears to be contradictory evidence regarding elevated cholesterol (total and low-density-lipoprotein) and a risk of thyroid eye disease [14]; however, elevated cholesterol does not appear to be the cause of thyroid eye disease, as the benefits of thyroid eye disease prevention do not become apparent with other (non-statin) anti-lipidemic agents [14-15]. Similarly, in our prior study, we used propensity-score matching to control for cholesterol [3]. Another hypothesis explaining the association between statin therapy and decreased development of thyroid eye disease includes reductions in insulin-like growth factor 1 and its respective signaling [6]. Others suggest that inhibition of mevalonate production is associated with induction of apoptosis, up-regulation of Th2 cells, mobilization of inflammatory T-cells away from the diseased site, reduced adipogenesis (involved with systemic inflammation), and anti-fibrosis activity in orbital myofibroblasts are further explanatory mechanisms [7, 9, 15, 16]. While it has been suggested that statins act synergistically with glucocorticoids (as demonstrated by the randomized controlled trial by Lanzolla and colleagues), this does not explain the physiology, as not all studies included corticosteroids [13]. While Lee et al. [9] suggest high-intensity statins have a greater protective effect (in women), Chou et al. [6] note that the protective effect does not vary across statin intensities. Furthermore, Nilsson et al. [6] demonstrate a protective effect across differing types of statins; in our previous study [3], we included all types of statins, showing an overall protective effect.

Limitations

Although many organizations have acknowledged that statins may be beneficial in thyroid eye disease prevention, none have included statins as an official recommendation in their current guidelines. Further research is needed to confirm the efficacy and safety of statins in thyroid eye disease. Limitations are evident in our study, for which data interpretation must be cautious. Mainly, only the CINAHL and MEDLINE databases were used. High heterogeneity between studies was present, suggesting the results of the primary studies may be too heterogeneous to be combined (including the background population of patients with Graves’ disease). Similarly, differences in study design, quality of studies, adjustment for confounders, and patient characteristics likely contribute to the heterogeneity. A particular fallacy includes the reliance on diagnostic claims/codes from administrative databases, which are prone to error and misclassification.

## Conclusions

In conclusion, this is the first meta-analysis to assess the impact of statin medications and the outcome of thyroid eye disease in patients with a diagnosis. We demonstrate a significantly reduced likelihood of developing thyroid eye disease with statin treatment; however, the explanation of this relationship remains unelucidated. Although our findings were statistically significant, high levels of heterogeneity between studies were present, limiting their overall interpretation and applicability. Further studies are needed with subgroup analyses to identify which patients are most likely to benefit from statin therapy.
